# Maternal F1 antibodies and cytokines in mother-neonate dog pairs in the *Marmota himalayana* plague focus

**DOI:** 10.1016/j.heliyon.2025.e42336

**Published:** 2025-01-30

**Authors:** Deming Tang, Asaiti Bukai, Shuai Qin, Ran Duan, Dongyue Lyu, Zhaokai He, Xiaojin Zheng, Weiwei Wu, Junrong Liang, Haifu Qu, Aidai Bieke, Peng Zhang, Dan Zhang, Haonan Han, Qun Duan, Huaiqi Jing, Xin Wang

**Affiliations:** a National Institute for Communicable Disease Control and Prevention, Chinese Center for Disease Control and Prevention, Beijing, China; b Dongcheng Center for Disease Control and Prevention, Beijing, China; c Akesai Kazak Autonomous County Center for Disease Control and Prevention, Jiuquan, China

**Keywords:** Plague, Dog, F1 antibody, Cytokines, Mother-to-child transmission

## Abstract

In this study, we investigated the F1 antibody against *Yersinia pestis* in the sera of mother-neonate shepherd dog pairs in the *Marmota himalayana* plague focus of the Altun-Qilian Mountains, Qinghai-Tibetan Plateau, China. Seropositive shepherd dogs lived in plague-endemic regions, where marmots were infected with *Y. pestis*, whereas seronegative dogs lived in non-endemic regions. The neonatal F1 antibody titers positively correlated with the maternal titers within 3 months after birth, and the neonatal titers were similar to or slightly lower than the maternal titers. In the absence of reinfection, antibodies in the neonates were obtained from their mothers; titers decreased with age and disappeared after 3 months. Mean tumor necrosis factor (TNF)-α, interleukin (IL)-6, IL-2, IL-10, and nerve growth factor (NGF)-β were higher in the mothers than in neonates. Maternal TNF-α, IL-10, vascular endothelial growth factor (VEGF)-A, and NGF-β and neonatal monocyte chemoattractant factor (MCP)-1 and VEGF-A were positively correlated with F1 antibody titers. Our results reveal continuing vertical transmission of F1 antibodies between mother dogs and their offspring and cytokine signatures under plague.

## 1 Introduction

Plague is a severe infectious disease caused by *Yersinia pestis* [1], which causes a series of severe clinical symptoms. According to the clinical manifestations, plague can be categorized as pneumonic, septicemic, bubonic, enteric, etc. [[1], [2], [3]]. The type Ⅲ secretion system allows *Y. pestis* to escape the immune system by preventing phagocytosis [4,5], causing infection. People infected with different strains of *Y. pestis* present various clinical symptoms and prognoses since different animal hosts and strain types can cause various immune responses [3,6,7].

Dogs are highly resistant to plague; only a few individuals develop plague after infection, and death is rare [8,9]. However, the mechanism underlying this high resistance is still unclear; some researchers believe it is related to the lack of N-formyl peptide receptors in dogs [10,11]. In addition, little is known about how maternal infection affects the immune system of the offspring. Shepherd dogs are indicators of plague foci, and monitoring the plague F1 antibody in these animals may reflect the intensity of the plague [12,13]. At birth, dogs primarily obtain immunoglobulin (Ig) G through passive immunity, which is facilitated by placental circulation and the ingestion of colostrum shortly after delivery [14]. Our previous research has shown that F1 antibodies decay rapidly in shepherd dogs (unpublished data); however, whether this rapid attenuation is related to the dog's high resistance to plague remains to be further studied.

Since severe *Y. pestis* infection causes a cytokine storm [15], different cytokine levels may reflect a certain immune state. Due to the complexity of each cytokine and its interactions, it has been suggested that measuring multiple cytokine concentrations for a given health condition will provide more relevant information than measuring a single cytokine [16,17]. As such, the cytometric bead array method can be used to assess the levels of multiple cytokines simultaneously, including monocyte chemoattractant factor (MCP)-1, nerve growth factor (NGF)-β, stem cell factor (SCF), vascular endothelial growth factor (VEGF)-A, interferon (IFN)-γ, tumor necrosis factor (TNF)-α, and interleukin (IL)-10, IL-12, IL-2, IL-6, and IL-8, with a small number of samples and high sensitivity.

The aims of this study were to (1) explore changes in the immune system of shepherd dogs after *Y. pestis* infection and (2) compare differences in cytokine levels among dogs with different F1 antibody titers. This study investigates the variation of plague F1 antibodies in mother dogs and their vertically transmitted offspring. To our knowledge, this is the first study to investigate the relationship between F1 antibody titers and cytokine levels. The results of this study might provide a foundation for future research on the assessment of cytokines to distinguish between infected and uninfected animals, improve our understanding of the maintenance of antibodies in shepherd dogs, and optimize the use of puppies as plague indicators.

## 2 Results

### 2.1 Distribution of shepherd dogs

In the *M. himalayana* plague-endemic and non-endemic areas in the Qinghai-Tibetan Plateau, we identified 97 shepherd dogs (18 mothers and 79 neonates) in 18 households, comprising 74 dogs (11 mothers and 63 neonates) in 11 households (A1–A11) in the Altun Mountains and 23 dogs (7 mothers and 16 neonates) in 7 households (S1–S7) in the Qilian Mountains (background information is available in Table S1). The neonates were born from December 2020 to March 2021. The initial sampling aimed to collect multiple times from puppies <1 month old. However, due to dispersed grazing and puppy relocation, only shepherd dogs from households A1–A10 achieved repeated sampling. Shepherd dogs from households S3 and S6, despite initial sampling of older puppies, two or more rounds were conducted to enhance data. Ultimately, dogs from 12 households (A1–A10, S3, and S6) were sampled twice or more (including the first and follow-up sampling), while all other dogs were sampled only once.

**Fig. 1:**
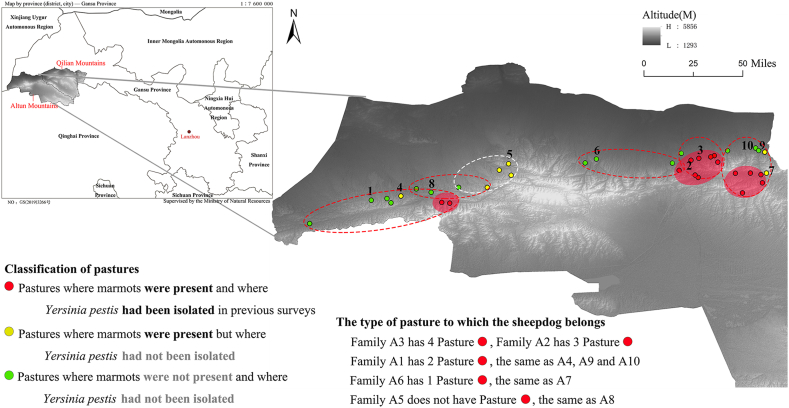
Distribution of pastures for shepherd dogs in the Altun Mountains Region, Tibetan Plateau, China. Numbers 1–10 represent pastures for households A1–A10, and ellipses/circles indicated by the dashed lines are the possible range of the shepherd dogs.

Throughout the year in 2020, the shepherd dogs moved with herders to different pastures, thus influencing their range of movement and exposure to *Y. pestis*, as shown in Fig. 1 by the pasture distribution of the shepherd dogs (households A1–A10) in the Altun Mountains. As shown in Fig. 1, shepherd dogs from households (1) A1, A4, and A8, (2) A5, (3) A2, A3, and A6, and (4) A7, A9, and A10 may have been infected with *Y. pestis* in 4 different regions, respectively. Only dogs from A5 were in pastures where *Y. pestis* had not been isolated in previous surveys (all were seronegative). In addition, the mean altitude of the pastures where marmots were not present (2841.37 m) was significantly (t = −3.93, *P* < 0.001) lower than that of the pastures where marmots were present (3234.00 m).

### 2.2 Variation of F1 antibody titers in shepherd dogs

Of the 210 total serum samples, 148 (70.48 %) were seropositive for F1 antibody; 124 (124/178, 69.66 %) serum samples from 11 households in the Altun Mountains and 24 (24/32, 72.73 %) serum samples from 7 households in the Qilian Mountains were seropositive. One hundred eighty-five samples (185/210, 88.10 %) were from shepherd dogs who underwent blood collection twice or more at different time periods. A total of 170 serum samples (170/210, 80.95 %) were collected during the marmot hibernation period (Table 1).

**Table 1:** Subject characteristics.

Mother dogs (n = 18)			Neonates (n = 79)				
Number of sample collections	N (%)	Geometric mean titer of F1 antibody	Household	N (%)	Age group at first blood sampling (days)	N (%)	Geometric mean titer of F1 antibody
4	1 (5.56)	289.92	A1	7 (8.86)	0–7	31 (39.24)	44.80
3	8 (44.44)		A2	7 (8.86)	8–14	16 (20.25)	
2	2 (11.11)		A3	5 (6.33)	15–30	21 (26.58)	
1	7 (38.89)		A4	3 (3.80)	31–70	11 (13.92)	
			A5	5 (6.33)			
			A6	7 (8.86)			
			A7	7 (8.86)			
			A8	6 (7.59)			
			A9	8 (10.13)			
			A10	6 (7.59)			
			Others	18 (22.79)			

**Fig. 2:**
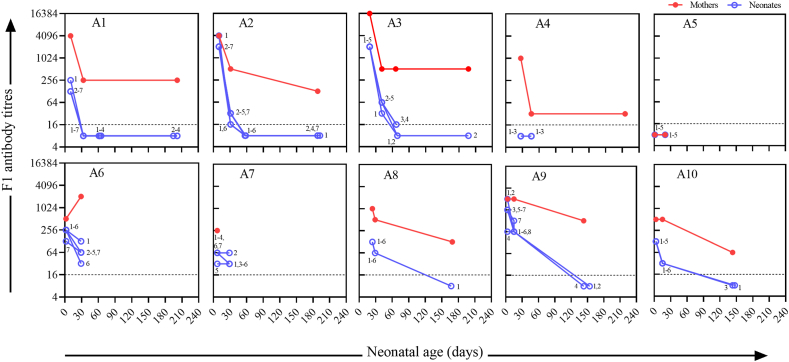
Variation in serum F1 antibody titers of mother-neonate pairs for households A1–A10. “⚪1″ refers to neonate no. 1 etc. from each household. A titer ≥1:16, indicated by the dashed line, was considered antibody-positive.

Of the 71 dogs (households A1–A10) from the Altun Mountains, 67 (9 mothers and 58 neonates) completed the 3-month follow-up (Fig. 2). The F1 antibody titer of the mother dog from A5 was seronegative throughout the study period, while that of the mother dog from A6 increased, and the rest was seropositive during the study. At the first blood collection, all neonates were seropositive except for 27-day-old and 5-day-old neonates from A4 and A5, respectively. The neonatal antibody titers gradually decreased with age (*r*_*s*_ = −0.733, *P* < 0.01). Nineteen of the 58 neonatal dogs exhibited a clear transition from seropositive to seronegative within 3 months. At about 6 months of age, all neonates were seronegative.

F1 antibody titers for shepherd dogs in the Qilian Mountains exhibited a similar pattern to that of the Altun Mountains (Fig. 2). The antibody titers of the neonatal dogs decreased rapidly within 1 month after birth. There is a statistically significant difference in the time required for the decrease of the F1 antibody titer between the newborns in households A1-A10 when divided into two groups according to the first sampling titer, which is either ≥1:2048 or <1:2048 (Kruskal–Wallis test, *P* < 0.05).

**Fig. 3:**
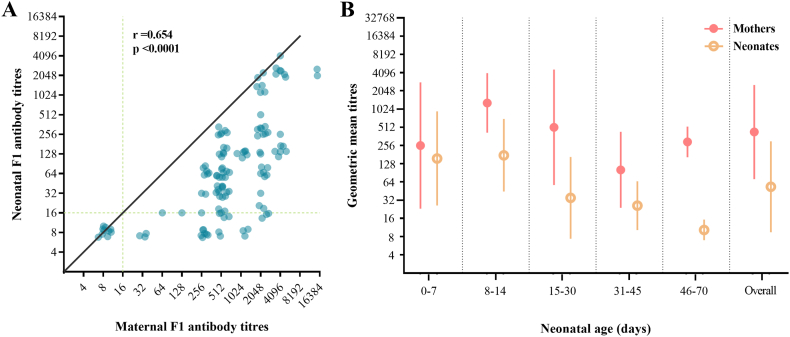
F1 antibody titers in mother-neonate pairs. (A) Correlation between antibody titers of neonates and mother dogs (overlap of data points was avoided; Spearman rank correlation coefficient *r*_*s*_ = 0.654, *P* < 0.0001). (B) Geometric mean titers for mother-neonate pairs by age group.

The scatter plot of the F1 antibody titers of mother-neonate pairs showed a positive correlation between pairs (*r*_*s*_ = 0.654, *P* < 0.0001) (Fig. 3). All points on the plot were below the line passing through its origin with a slope of 1, indicating that the titers of the neonatal dogs were never higher than that of their mothers (Fig. 3A). The points only intersected the line at high and low titers, as neonatal dogs from A2 and A9 had the same titers as their mothers at the initial test and the dogs from A5 were all seronegative. The geometric mean of titers for both mother and neonatal dogs decreased with age (Fig. 3B).

### 2.3 Western blotting of serum F1 antibodies from shepherd dogs using the F1 antigen

**Fig. 4:**
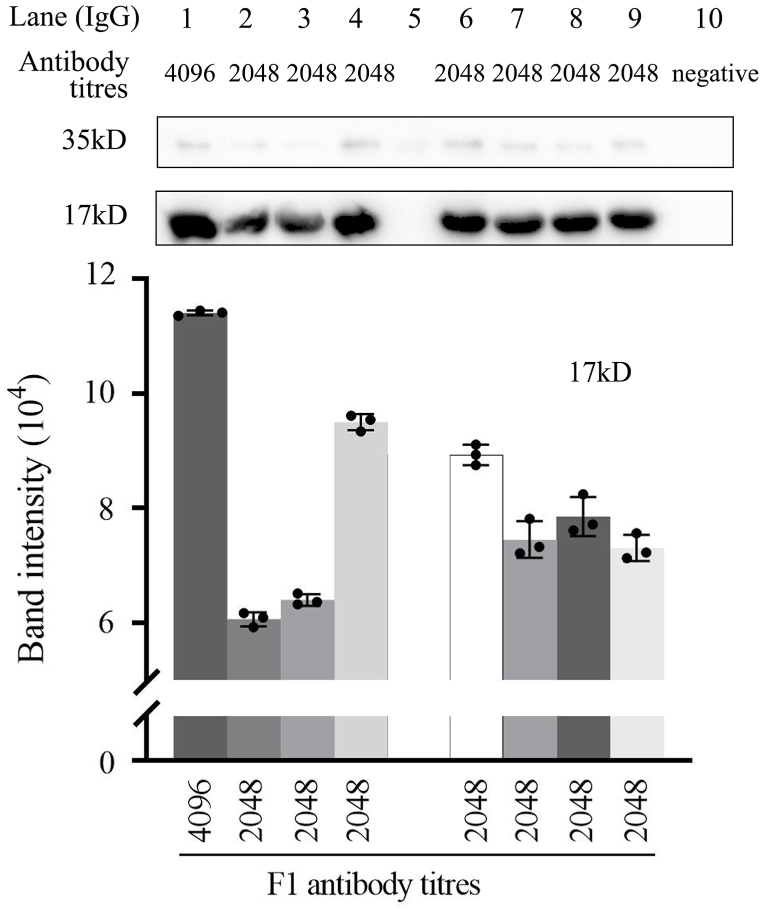
Western blot analysis of the F1 antigen and positive serum antibody in shepherd dogs (the full, non-adjusted images of gel and blot are shown in Figs. S2 and S3, respectively).

Western blotting was used to detect F1 IgG and IgM in the mother-neonate pairs. Fig. 4 shows the bands obtained after binding the F1 antigen in 9 serum samples with various F1 antibody titers, using rabbit anti-dog IgG or goat anti-dog IgM as secondary antibodies. For both mother and neonatal dogs, western blotting identified 2 protein bands (17 and 35 kDa) as highly immunoreactive. The relative molecular weight of the F1 antigen was ∼12–17 kDa; the relative molecular weight of the 4 subunits was used to confirm these were the target bands. As the titer level decreased, the integrated optical density (IOD) of the bands decreased. IgM was only found in the mother dog from A6.

### 2.4 Dynamics of cytokine levels and relationships with F1 antibody titers

To explore cytokine levels, 184 serum samples from follow-up dogs were assessed. Dogs were grouped according to their F1 antibody results to present their cytokine characteristics (seropositive/seronegative), and neonates were further grouped by age.

**Fig. S1:**
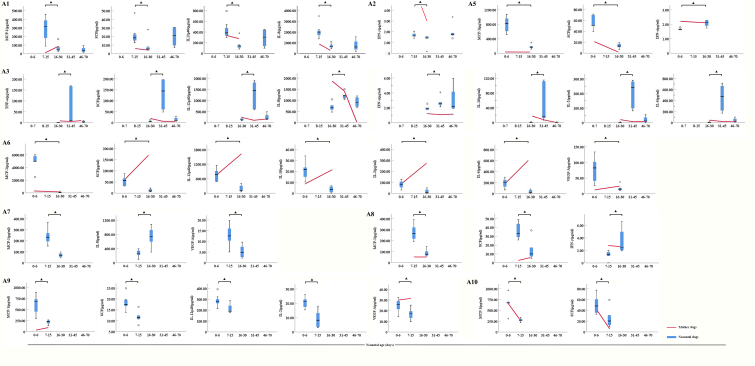
Levels of cytokines in dogs in each household.

Twenty-five and 2 serum samples were detected as M+ and M−, respectively, whereas 147 and 10 serum samples were classified as N+ and N− for the first blood collection, respectively. In the N+ group, seropositive rates in subgroups 0–7, 8–14, 15–30, 31–45, and 46–70 days were 100 % (19/19), 100 % (41/41), 78.00 % (39/50), 70.00 % (7/10), and 33.33 % (9/27), respectively. Fig. S1 shows the cytokine dynamics in mother-neonate pairs by household.

**Fig. 5:**
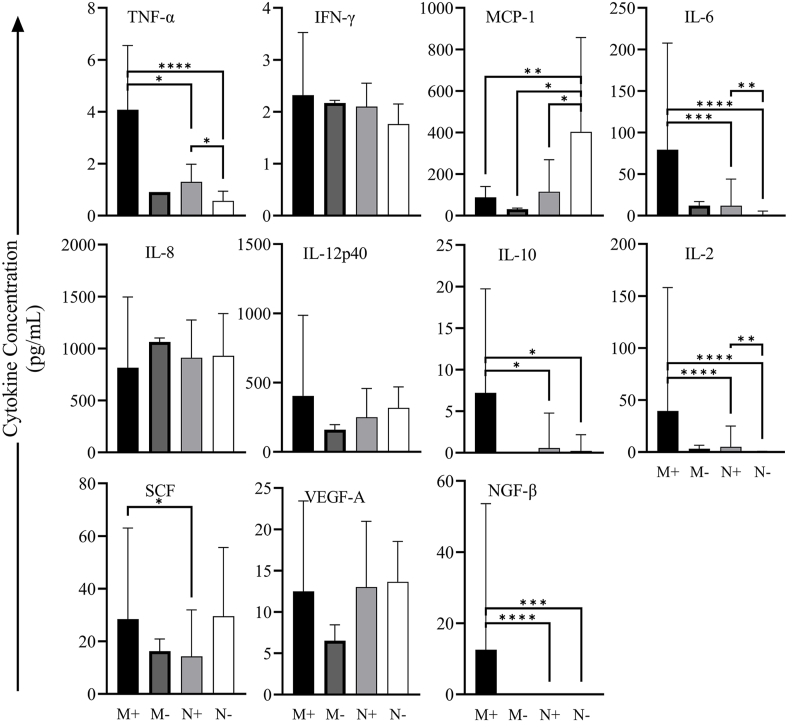
Comparison of cytokine levels in mother and neonatal dogs. Significant differences (connecting lines) are denoted as ∗*P* < 0.05; ∗∗*P* < 0.005; ∗∗∗*P* < 0.0005; ∗∗∗∗*P* < 0.0001.

**Fig. 6:**
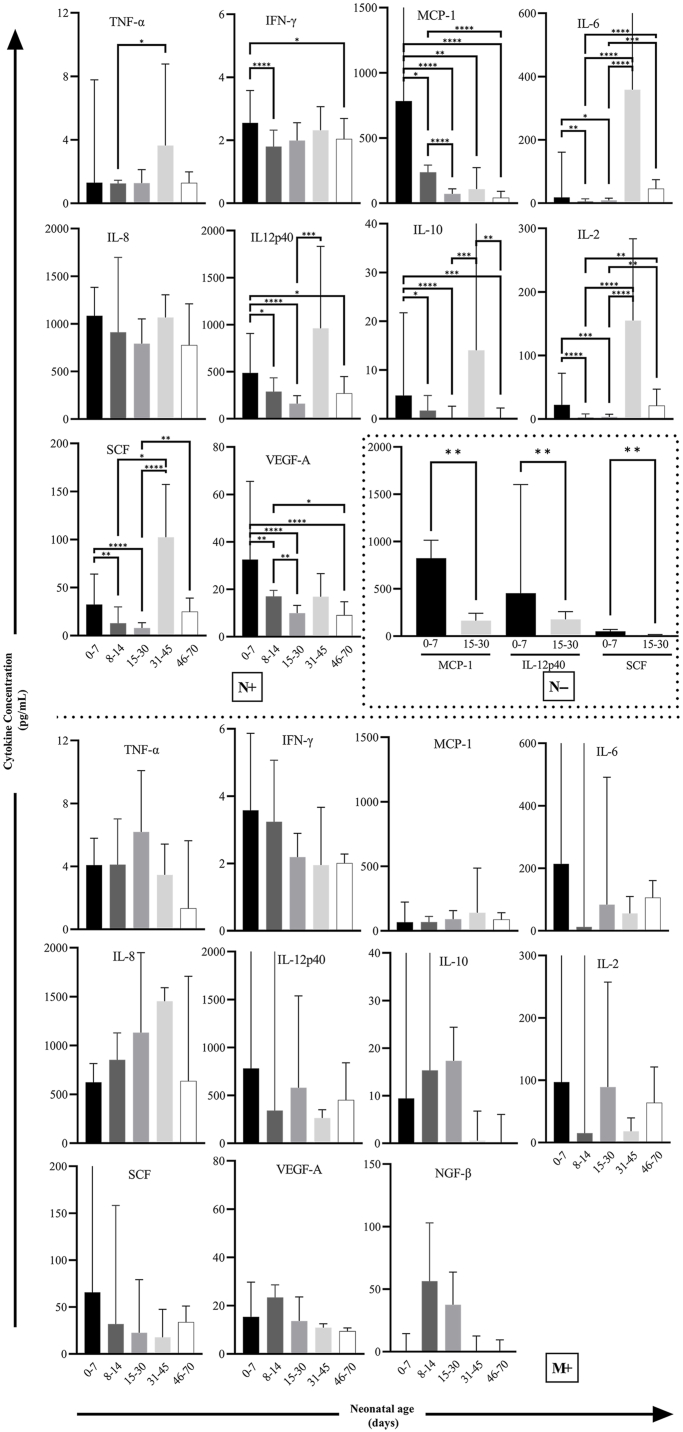
Changes in cytokine concentrations in dogs at different ages.

Differences in cytokines levels between mother and neonatal dogs are shown in Fig. 5. TNF-α, IL-6, IL-10, IL-2, and NGF-β were higher in the M+ group than in the N+ or N− group, and TNF-α, IL-6, and IL-2 were higher in the N+ group than in the N− group. MCP-1 was higher in the N− group than in the other groups. No significant differences in cytokine levels were observed among the different neonatal age subgroups for the M+ group. In contrast, the N+ group had higher cytokine levels for subgroups 0–7 and 31–45 days (with an increase in cytokine levels for the latter age subgroup) except for IL-8 and NGF-β. In the N+ group, MCP-1 and VEGF-A peaked at 0–7 days and then decreased with age. IL-6, IL-12p40, IL-10, IL-2, and SCF decreased for the first 30 days and then increased at 31–45 days. In the N− group, only serum samples of neonates at 0–7 and 15–30 days were collected, and MCP-1, IL-12p40, and SCF decreased with age (Fig. 6). NGF-β levels in the N+ and N− groups were extremely low and could not be detected.

**Fig. 7:**
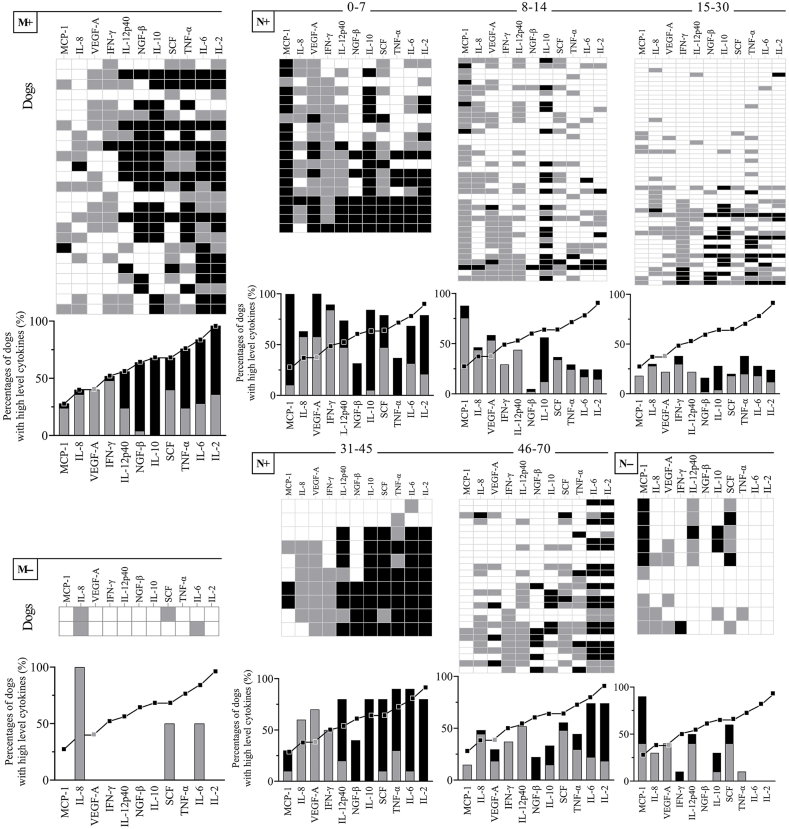
Overall signature of high cytokine levels in the different dog groups. The high proportion of high and extremely high Cytokine Indices in the M+ group was used to generate the reference cytokine signature curves () to identify changes in the overall cytokine signature in all other groups.

The Cytokine Index was calculated to further characterize the overall cytokine signatures of mother and neonatal dogs, which classified samples into low, high, and extremely high categories. Comparative analysis of the cytokine signatures, using the M+ curves as a reference, demonstrated that the M+ group generally had higher cytokine levels than the M− group. The proportion of high and extremely high MCP-1 Cytokine Index in the N+ group decreased with age from 0 to 30 days, and rised rises to the level of the M+ group in 31–45 days (Fig. 7). The proportion of high and extremely high IL-2 Cytokine Index was highest in seropositive dogs (96.0 % for M+, 56.28 % for N+, and 0 % for both M− and N−).

**Fig. 8:**
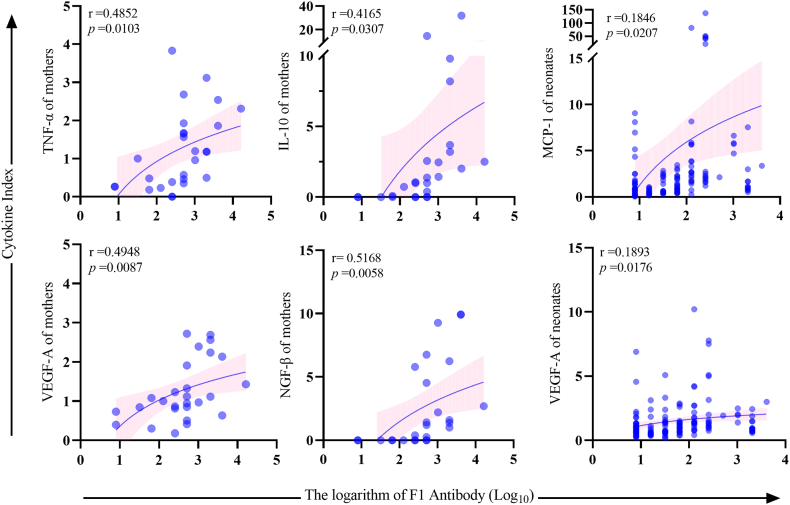
Correlations of Cytokine Indices with F1 antibody titers.

Scatter plots of logarithmic fitted curves of the Cytokine Indices and F1 antibody titers are shown in Fig. 8. The F1 antibody titers were found to positively correlate with TNF-α (r = 0.4852, *P* = 0.0103), IL-10 (r = 0.4165, *P* = 0.0307), VEGF-A (r = 0.4948, *P* = 0.0087), and NGF-β (r = 0.5168, *P* = 0.0058) in mother dogs, and with MCP-1 (r = 0.1846, *P* = 0.0207) and VEGF-A (r = 0.1893, *P* = 0.0176) in neonatal dogs.

## 3 Discussion

This study describes for the first time the relationship of serum F1 antibodies and cytokine levels between shepherd mother dogs and their offspring with high plague resistance in *M. himalayana* plague-endemic and non-endemic areas in the Qilian-Altun Mountains, Tibetan Plateau. In this natural plague focus, marmots are closely related to shepherd dogs and are the main host of *Y. pestis* [18]; however, their movements decrease after infection with *Y. pestis*. Shepherd dogs can easily capture infected marmots, but as highly plague-resistant animals, most shepherd dogs can survive and produce F1 antibodies after being infected with *Y. pestis*.

Here, the distribution and changes in antibody titers of shepherd dogs from households A1–A10 showed that (1) During the 0–2 month age period, antibodies are primarily derived from passive immunization through vertical transmission, while their immune response and acquired immunity are not yet fully developed [19]. The maternal antibody IgG levels in neonates from A1 and A2 show a declining trend around days 10 and 30 (Fig. 2 and Table S1). The exposure rate of *Y*. *pestis* in the pastures of A2 was higher than that of A1, and the maternal antibodies in A2 neonates from high exposure areas declined more rapidly. This is similar to the decline observed in maternally derived immunoglobulin G antibodies to both the EBV viral capsid antigen and EBV nuclear antigen [20]. We speculate that F1 antibodies, which serve as protective antibodies, may be consumed by neutralizing responses to pathogen antigens upon contact with an infectious agent. We cannot exclude the possibility that the A2 neonates were exposed to infection sources more frequently, which accelerated the titer depletion *in vivo*. (2) Household A3 had the highest plague prevalence and F1 antibody titer among the mother dogs, whereas household A5 had the lowest plague prevalence (*Y. pestis* was never isolated), and the A5 neonates were seronegative from day 1 after birth. (3) The A4 mother dog was seropositive, while the neonates were seronegative. Since the antibody titer of the mother dog as the primary variable/factor for assessing the passive antibody level, and considering that the neonatal immune system is less developed than that of the mother—with the latter's immunity unable to stabilize maternal antibody levels due to the absence of immune memory—we speculate that the neonates had converted from seropositive to seronegative before the first blood sampling (27 days after birth). (4) The A6 mother dog was the only dog with an increased titer, presumed to be newly infected Tibetan Mastiff. (5) Household A8 was in the same non-endemic area as household A5, but we believe the dogs became infected because A8 was closer to an endemic area (Fig. 1). (6) In the follow-up where mother-neonate pairs were sampled, the F1 antibody titers were mainly recorded from birth to 3 months, which showed a dynamic change in titers of the shepherd dogs and the transfer of maternal F1 antibodies.

Based on the results, we infer that when dogs are not preying on marmots, resulting in reinfection, the F1 antibody titers of neonatal dogs would be similar to or slightly lower than those of their mothers. The titers of the neonatal dogs gradually decreased with age and then steeply declined within 1 month. Our results are consistent with those by Winters et al. [21], showing that antibodies against the Rift Valley fever virus, canine distemper virus, and infectious canine hepatitis virus were transferred from mothers to neonates *in utero* and through breastfeeding, and the titers of these antibodies in the serum of neonatal dogs decreased with age. The difference in the F1 antibody titers between mother and neonatal dogs at birth may be related to the placental and intestinal barriers, as the placental endothelial chorion only allows 5 %–10 % of maternal antibodies to be transferred to the fetus, and the placental barrier only transmits IgG and blocks IgM [22,23]. Western blot analysis indicated that there were other antibody types (e.g., IgM) in the mother dogs besides IgG, while only IgG occurred in the neonatal dogs. In addition, early colostrum intake may optimize intestinal passive immune transfer of IgG [[24], [25], [26]]. The duration of passive immunity of puppies depends on the antibody titer level of the mother and the maternal derived antibody half-life. While we observed that the time required for the F1 antibody titer to decrease was relatively short for pups with titers ≥1:2048, this finding must be considered in conjunction with various factors, such as surrounding environment, growth rate, and breed, to reach a more accurate conclusion.

As the samples in this study were mostly collected during the hibernation period of marmots, the possibility of dogs being exposed to *Y. pestis* was very low (only the titer of mother dog A6 increased, as shown in Fig. 1). Generally, maternal antibody levels in the neonatal dogs typically peak following the ingestion of breast milk and subsequently decline naturally in the absence of further exposure to infection [26]. Neonatal dogs that are 3 months old can already eat marmots and are more likely to be exposed to natural infection. Therefore, antibodies in neonatal dogs aged >3 months may be acquired from natural infection rather than from the mother. Based on the titers of mother-neonate pairs in this period (when neonates were older than 3 months) and the slower F1 antibody decay in mother dogs compared with neonatal dogs, antibodies were maintained for a longer time in mother dogs and neonates aged >3 months (Fig. 3). The reason for this phenomenon may be related to the difference in blood volume and the mechanism of *Y. pestis* resistance in *Canidae*. Research has shown that while the blood volume of adult dogs is relatively constant, the blood volume of neonates increases rapidly after birth [27], which may lead to the dilution of antibodies. It is unknown whether the F1 antibody titer decreases more slowly in canines than in low-resistant animals, and the detailed mechanism of canine resistance to *Y. pestis* remains to be investigated.

During *Y. pestis* infection, cytokines are produced by immune cells to trigger an inflammatory reaction, which is crucial to eliminate the pathogen. However, excessive secretion of cytokines in a highly inflammatory state may trigger a cytokine storm, resulting in uncontrolled inflammation in tissues and critical organs. There are many studies on the roles of various cytokines in the pathogenesis of plague, although the data are inconsistent. Maternal *Y. pestis* infection during pregnancy has been shown to have permanent and tissue-specific effects on the immunity of offspring [28]. In this study, the levels and dynamics of 11 cytokines in shepherd dogs were assessed, which showed that mother dogs infected with *Y. pestis* had higher levels of these cytokines than neonatal dogs.

Several cytokine levels in the N+ group generally decreased in the subgroups 0–7, 8–14, and 15–30 days but increased for 31–45 days. This trend may be related to cytokine secretion volume, immature growth and development, or vertical transmission in neonatal dogs. Dramatic decreases in TNF-α and IFN-γ are characteristic of *Y. pestis* infection, as the V antigen of *Y. pestis* can indirectly or directly downregulate TNF-α and IFN-γ. Most studies have shown that LcrV upregulates the anti-inflammatory cytokine IL-10 and thus inhibits the production of TNF-α and IFN-γ in cells [29,30]. TNF-α and IFN-γ have been shown to prevent the loss of function or death of mice infected with *Y. pestis*. In this study, the non-downregulation of TNF-α and IFN-γ in dogs may be one of the reasons for the high resistance against *Y. pestis*.

There was a higher proportion of dogs with a high and extremely high IL-2 Cytokine Index in the N+ group than in the N− group. IL-2, also known as T cell growth factor, stimulates T cell activation and proliferation and induces T cells and natural killer cells to produce IFN-γ, TNF-β, and other factors. A high level of IL-2 may be an immune feature or key to *Y. pestis* infection. The proportion of high and extremely high IL-6 Cytokine Index was also greater in the M+ group. IL-6 plays a role in acute inflammatory response mainly by its proinflammatory effect on various cells and by inducing the production of acute reactive protein in the liver. After 24–48 h of *Y. pestis* infection, YopM upregulates IL-6 [31]. Maternal IL-6 produced in response to infection can directly cause epigenetic changes in fetal intestinal epithelial stem cells, leading to long-lasting impacts on intestinal immune homeostasis [28].

MCP-1 upregulates adhesion factors on monocytes and induces the secretion of IL-1β and IL-6. Epithelial cell production of MCP-1 is one of the ways in which hosts maintain an early response to *Yersinia* [32]. MCP-1 levels were similar in the N+ and N− groups at 0–7 days, which had the highest MCP-1 level compared with the other subgroups in neonates and mother dogs (Fig. 6). Based on the cytokine signature (Fig. 7), it appears that the cytokine levels in the N+ group decreased with age from 0 to 30 days, whereas levels increased from 31 to 45 days, having a similar cytokine profile to that of the M+ group. Similar to the increase in cytokines induced by the biovar Microtus strain of *Y. pestis* [33], the secretion of Th1- (IL-2 and TNF-α) and Th2-related cytokines (IL-6 and MCP-1) increased in infected subjects, but there were no significant differences in IFN-γ or IL-8.

MCP-1 and VEGF-A in neonates peaked at 0–7 days and then decreased with age. Although there was a positive correlation between F1 antibody titer with MCP-1 and VEGF-A, a decrease in their concentrations at different ages so that MCP-1 and VEGF-A were correlated with the birth status (0–7 days of age). Furthermore, based on the functions of maternal and neonatal cytokines that are positively correlated with F1 antibody titer, TNF-α, MCP-1, and VEGF-A may be closely related to the presence of F1 antibody.

## 4 Materials and methods

### 4.1 Selection of study areas and collection of specimens

We investigated shepherd dogs in the *Marmota himalayana* plague-endemic and non-endemic areas in the Altun-Qilian Mountains of the Qinghai-Tibetan Plateau, China. The study areas were classified into 3 categories based on the presence of marmots in the pastures and whether *Y. pestis* had been isolated from the pastures in previous surveys: (1) pastures where marmots were present and *Y. pestis* had been isolated; (2) pastures where marmots were present and *Y. pestis* had not been isolated; and (3) pastures where marmots were not present and *Y. pestis* had not been isolated. Shepherd dogs that lived with herders and had given birth or had been born ≤3 months prior were selected as the research subjects. Mothers and their pups who met these inclusion criteria were also included in the follow-up cohort. In the Altun Mountains, for neonates less than one month old during the initial sampling, we conducted two or more sampling sessions for both the neonatal dogs and their mothers, while all other dogs were sampled only once. Additionally, we included follow-up data on eligible neonates and their mothers from our previous study (unpublished data) in the Qilian Mountains. Out of a total of 97 sheepdogs, 61 neonates and 11 mother dogs underwent multiple sample collections, resulting in the acquisition of two or more blood samples at different collection times.

Blood collection mainly occurred during the marmot hibernation period from October to April of the following year. Blood was collected from the leg veins of the mother dogs and their neonates into 5-mL vacuum blood collection tubes. During the blood collection process, each dog was pacified to ensure safety. The date of birth of the pup, blood collection date, and location and altitude of the pastures that the mother herded at different times of the year were collected (Table S1).

Seropositive and seronegative F1 antibody results in mother dogs were designated M+ and M−, respectively, and seropositive and seronegative F1 antibody results in the first blood collection of neonates were designated N+ and N−, respectively. Various animal studies have shown that the half-life of maternal antibodies in newborn animals is < 7 days [24,26]. Therefore, N+ samples were grouped into 0–7, 8–14, 15–30, 31–45, and 46–70 days, while N− samples were grouped into 0–7 and 15–30 days.

### 4.2 Indirect hemagglutination assay

F1 antibody titers were measured by serial dilution of serum samples using the microtiter plate method and Plague F1 Antibody Test Kit (developed by Qinghai Institute for Endemic Disease Prevention and Control, Xining, Qinghai Province, China). F1 antigen inhibition controls, negative controls, and positive controls were also established. An antibody titer ≥1:16 was defined as seropositive.

### 4.3 Western blotting

Hybridization against plague F1 antigen was confirmed by Western blotting. Protein was extracted from F1 antibody-positive serum samples from shepherd dogs, quantified, separated by sodium dodecyl sulfate-polyacrylamide gel electrophoresis, and transferred to a membrane to hybridize against the F1 antigen. Rabbit anti-dog IgG and goat anti-dog IgM were used as secondary antibodies. The IOD values of the bands were measured using ImageJ.

### 4.4 Cytometric bead array

Using a Cytokine/Chemokine/Growth Factor 11-Plex Canine ProcartaPlex™ Kit 1 (EPX11A-50511-901; Invitrogen, Waltham, MA, USA), 11 canine cytokines (SCF, IFN-γ, IL-2, IL-6, IL-8, IL-10, IL-12p40, MCP-1, VEGF-A, TNF-α, and NGF-β) were assessed on a Luminex 200 instrument. Analyte concentrations were determined using a 5-parameter logistic, nonlinear regression curve calculated from 7 standards. The lower detection limit was based on the standard curve generated at the time of detection.

### 4.5 Cytokine profile analysis

The overall median of each cytokine was used as the cut-off value to distinguish between the low and high levels of each cytokine [34]. The Cytokine Index was defined as the ratio of the cytokine concentration to the median cytokine concentration and was divided into the following categories: low: 0 < Cytokine Index <1; high: 1 < Cytokine Index <3; extremely high: Cytokine Index ≥3. The proportion of shepherd dogs with a high Cytokine Index was calculated as proportion = (high + extremely high)/total × 100 %. Radar charts were further used to summarize the cytokine signatures in each group; the highest proportions of high and extremely high Cytokine Indexes in the M+ groups were used as the reference to identify changes in the overall cytokine patterns in all other groups.

### 4.6 Statistical analysis

The F1 antibody titers are presented as the geometric mean for mother and neonate dog samples; differences between means were compared in a logarithmic scale. Cytokine concentrations are presented as medians with interquartile ranges; values below the lower detection limit were regarded as zero. The hemolysis data for the blood samples were discarded. The F1 antibody titers and cytokine levels were non-normally distributed; therefore, the Kruskal–Wallis test was used to analyze the differences in F1 antibody titers and cytokine levels among dogs, and Dunnett's test was used for pairwise comparisons. Spearman correlation analysis was used to analyze the relationship between F1 antibody titers and cytokine levels. A 2-sided *P*-value <0.05 was considered statistically significant.

## CRediT authorship contribution statement

**Deming Tang:** Writing – review & editing, Writing – original draft, Visualization, Validation, Software, Investigation, Data curation. **Asaiti Bukai:** Writing – review & editing, Writing – original draft, Methodology, Investigation, Formal analysis, Data curation. **Shuai Qin:** Writing – review & editing, Writing – original draft, Validation, Investigation, Data curation. **Ran Duan:** Writing – review & editing, Writing – original draft, Validation, Investigation, Data curation. **Dongyue Lyu:** Writing – review & editing, Writing – original draft, Validation, Investigation. **Zhaokai He:** Writing – review & editing, Writing – original draft, Validation, Investigation. **Xiaojin Zheng:** Writing – review & editing, Resources, Project administration, Methodology. **Weiwei Wu:** Writing – review & editing, Investigation. **Junrong Liang:** Writing – review & editing, Validation. **Haifu Qu:** Writing – review & editing, Investigation. **Aidai Bieke:** Writing – review & editing, Investigation. **Peng Zhang:** Writing – review & editing, Investigation. **Dan Zhang:** Writing – review & editing, Investigation. **Haonan Han:** Writing – review & editing, Investigation. **Qun Duan:** Writing – review & editing, Investigation. **Huaiqi Jing:** Writing – review & editing, Supervision, Resources, Project administration, Funding acquisition, Formal analysis, Conceptualization. **Xin Wang:** Writing – review & editing, Supervision, Resources, Project administration, Methodology, Funding acquisition, Formal analysis, Conceptualization.

## Ethics statement

The animal experiments involved in this study were approved by the Laboratory Animal Welfare & Ethics Committee (the animal ethics approval number: 2020-003) in accordance with ethical principles and the study complies with all regulations.

## Data availability statement

Data included in the supplementary material.

## Funding

This work was supported by the 10.13039/501100012166National Key Research and Development Program of China 2022YFC2602203.

## Declaration of competing interest

The authors declare that they have no known competing financial interests or personal relationships that could have appeared to influence the work reported in this paper.

## Figures and Tables

**Fig. S2:**
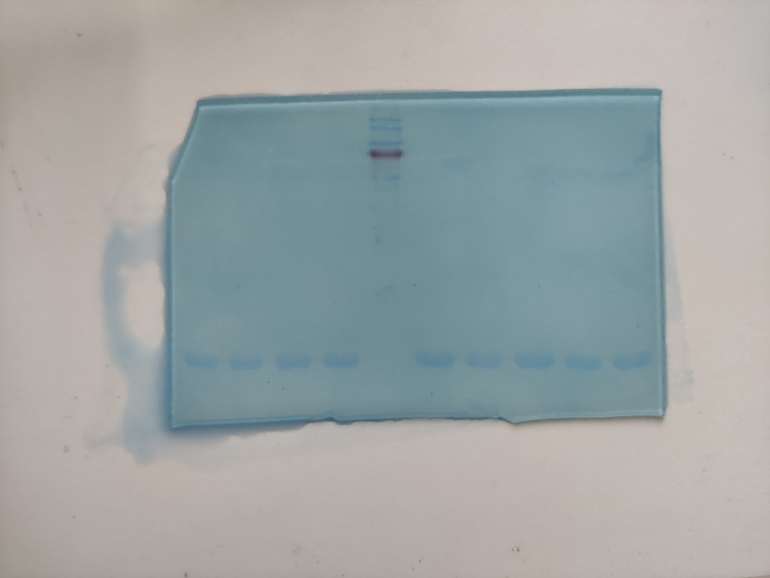
Uncropped gel image corresponds to Fig. 4. The gel has been electrotransferred and stained with Coomassie brilliant blue.

**Fig. S3:**
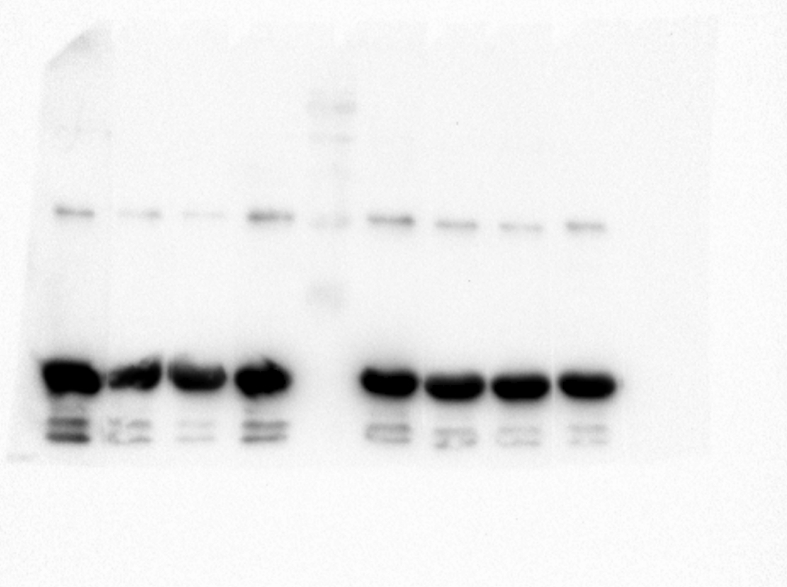
Uncropped blot image corresponds to Fig. 4.
