# Supplementary material for: Maternal F1 antibodies and cytokines in mother-neonate dog pairs in the Marmota himalayana plague focus
Source: Heliyon. 2025 Jan 30;11(3):e42336. doi: 10.1016/j.heliyon.2025.e42336 (PMC11847103; doi:10.1016/j.heliyon.2025.e42336)
Supplement: Multimedia component 1 [file mmc1.pdf]

Table S1:Blood sample collection and pasture information for each household

Altun Mountain region A1

Species	shepherd dog	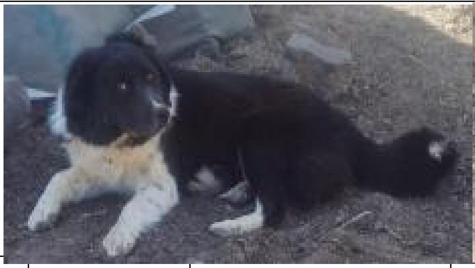 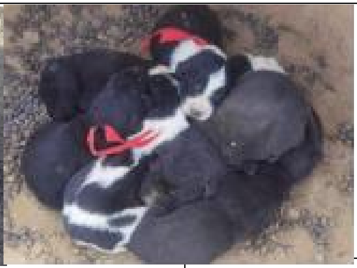
Pastures		

Seasons	Location	Altitude	Latitude and longitude	Distribution of marmot	Detection of *Y. pestis*
①Spring	Annanba	3198m	93°8'28.99"E 39°14'2.70"N	abundance	first detected in 1983
②Summer	Denggaike	3319m	93°10'43.60"E 39°13'49.73"N	abundance	first detected in 1983
③Autumn	Kushuihe	2746m	92°31'5.09"E 39°8'14.76"N	nonexistent	—
④Winter	Jiaertasi	3026m	92°48'30.25"E 39°14'36.89"N	nonexistent	—

NO.	Date of birth	Sampling date and Pasture number	F1 antibody titer	Sampling date and Pasture number	F1 antibody titer	Sampling date	F1 antibody titer	Sampl-ing date	F1 antibody titer
A20D1 mother	—	2020.10.27 ③	1:4096	2020.11.19 ④	1:256	/		2021.5.7	1:256
A20D1-1	2020.10.17		1:256		negative	2020.12.17	negative	/	
A20D1-2			1:128		negative	2020.12.18	negative	2021.5.7	negative
A20D1-3			1:128		negative		negative		negative
A20D1-4			1:128		negative	2020.12.21	negative	2021.4.30	negative
A20D1-5			1:128		negative	/		/	
A20D1-6			1:128		negative	/		/	
A20D1-7			1:128		negative	/		/	

A2

Species	shepherd dog	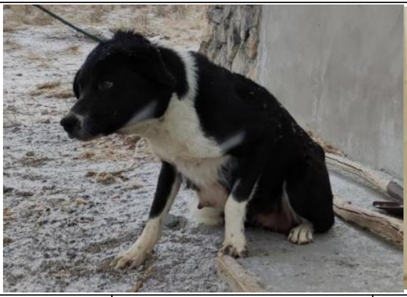 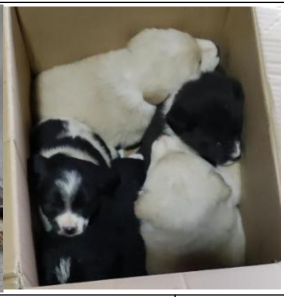
Pastures		

Seasons	Location	Altitude	Latitude and longitude	Distribution of marmot	Detection of *Y. pestis*
①Spring	Bangewa	2800m	94°25'40.7"E 39°26'48.33"N	few	detected
②Summer	Bangewa	3311m	94°26'39.01"E 39°25'01.85"N	abundance	detected
③Autumn	The national highway 215	2382m	94°16'22.44"E 39°27'27.84"N	nonexistent	—
④Winter	Sangequanzi	2763m	94°19'3.60"E 39°25'33.56"N	existent	detected

NO.	Date of birth	Sampling date and Pasture number	F1 antibody titer	Sampling date and Pasture number	F1 antibody titer	Sampling date	F1 antibody titer	Sampl-ing date	F1 antibody titer
A20D2 mother	—	2020.10 .30 ④	1:4096	2020.11 .20 ④	1:512	/		2021 .4.26	1:128
A20D 2-1	2020. 10.20		1:4096		1:16	2020.12. 16	nega-tive	2021 .4.30	nega-tive
A20D 2-2			1:2048		1:32		nega-tive	2021 .4.26	nega-tive
A20D 2-3			1:2048		1:32		nega-tive	/	
A20D 2-4			1:2048		1:32		nega-tive	2021 .4.26	nega-tive
A20D 2-5			1:2048		1:32	2020.12. 17	nega-tive	/	
A20D 2-6			1:2048		1:16		nega-tive	2021 .4.26	nega-tive
A20D 2-7			1:2048		1:32		nega-tive	/	

A3

Species	shepherd dog	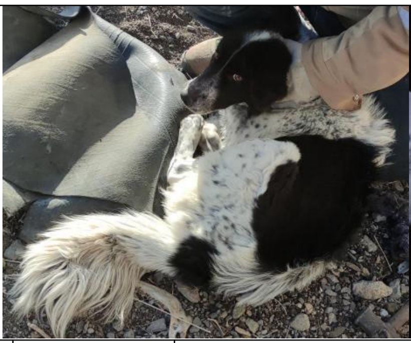 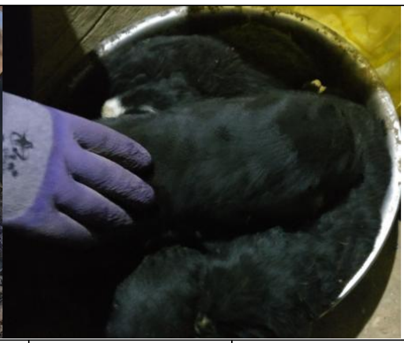
Pastures		

Seasons	Location	Altitude	Latitude and longitude	Distribution of marmot	Detection of *Y. pestis*
①Spring	Bangewa	2850m	94°24'38.50"E 39°26'27.75"N	few	detected
②Summer	Changcaogou	3124m	94°21'5.08"E 39°20'50.34"N	existent	detected
③Autumn	Changcaogou	3086m	94°20'14.13"E 39°21'31.21"N	existent	detected
④Winter	Sangequanzi	2773m	94°21'16.21"E 39°26'04.70"N	existent	detected

NO.	Date of birth	Sampling date and Pasture number	F1 antibody titer	Sampling date and Pasture number	F1 antibody titer	Sampling date	F1 antibody titer	Sampl-ing date	F1 antibody titer
A20D3 mother	—	2020.10.30 ③	1:16384	2020.11.21 ④	1:512	2020.12.16	1:512	2021.4.26	1:512
A20D3-1	2020.10.11		1:2048		1:32	2020.12.19	nega-tive	/	
A20D3-2			1:2048		1:64		nega-tive	2021.4.26	nega-tive
A20D3-3			1:2048		1:64	2020.12.17	1:16	/	
A20D3-4			1:2048		1:64		1:16	/	
A20D3-5			1:2048		1:64	/		/	

A4

<table>
<tr><td>Species</td><td>shepherd dog</td><td rowspan="2">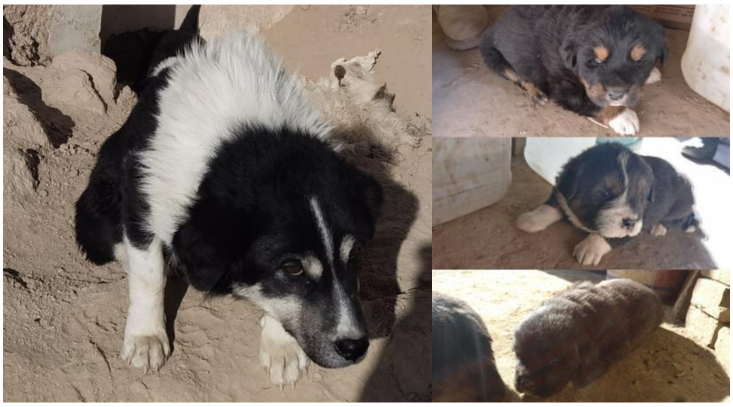</td></tr>
<tr><td colspan="2">Pastures</td></tr>
</table>

Seasons	Location	Altitude	Latitude and longitude	Distribution of marmot	Detection of *Y. pestis*
①Spring	Annanba	3027m	93°6'10.05"E 39°15'0.59"N	abundance	first detected in 1983
②Summer	Denggaike	3343m	93°10'58.61"E 39°13'42.43"N	abundance	first detected in 1983
③Autumn	Jiuquannon gkan	2916m	92°56'55.37"E 39°15'45.18"N	existent	not detected
④Winter	Jiaertasi	3154m	92°49'17.68"E 39°14'11.09"N	nonexistent	—

<table>
<tr><th>NO.</th><th>Date of birth</th><th>Sampling date and Pasture number</th><th>F1 antibody titer</th><th>Sampling date and Pasture number</th><th>F1 antibody titer</th><th>Sampling date</th><th>F1 antibody titer</th></tr>
<tr><td>A20D4 mother</td><td>—</td><td rowspan="4">2020.10.31
③</td><td>1:1024</td><td rowspan="4">2020.11.21
④</td><td>1:32</td><td>2021.5.7</td><td>1:32</td></tr>
<tr><td>A20D 4-1</td><td rowspan="3">2020.10.4</td><td>nega-tive</td><td>nega-tive</td><td colspan="2">/</td></tr>
<tr><td>A20D 4-2</td><td>nega-tive</td><td>nega-tive</td><td colspan="2">/</td></tr>
<tr><td>A20D 4-3</td><td>nega-tive</td><td>nega-tive</td><td colspan="2">/</td></tr>
</table>

A5

Species	shepherd dog	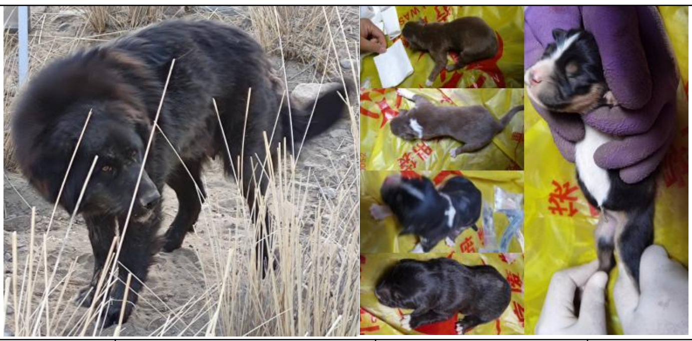
Pastures		

Seasons	Location	Altitude	Latitude and longitude	Distribution of marmot	Detection of *Y. pestis*
①Spring	Heziletasi	3109m	93°24'46.85"E 39°22'52.21"N	existent	not detected
②Summer	Kuerlemike	3510m	93°28'07.58"E 39°21'27.16"N	existent	not detected
③Autumn	Qingshigou	3138m	93°27'24.71"E 39°24'35.52"N	few	not detected
④Winter	Saersayi	3315m	92°54'05.91"E 39°13'55.70"N	nonexistent	—

NO.	Date of birth	Sampling date and Pasture number	F1 antibody titer	Sampling date and Pasture number	F1 antibody titer
A20D5 mother	—	2020.10.31 ③	negative	2020.11.19 ③	negative
A20D 5-1	2020.10.30		negative		negative
A20D 5-2			negative		negative
A20D 5-3			negative		negative
A20D 5-4			negative		negative
A20D 5-5			negative		negative

A6

<table>
<tr><td>Species</td><td>Tibetan Mastiff</td><td rowspan="2">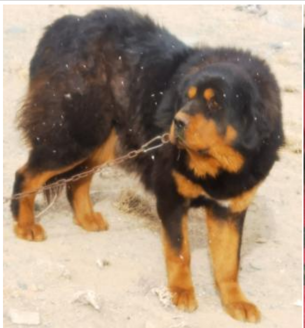 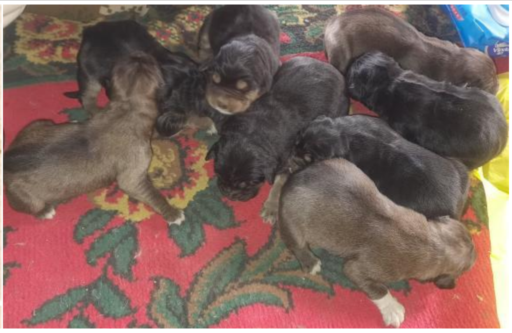</td></tr>
<tr><td colspan="2">Pastures</td></tr>
</table>

Seasons	Location	Altitude	Latitude and longitude	Distribution of marmot	Detection of *Y. pestis*
①Spring	Dalebai	2602m	94°13'48.25"E 39°24'47.11"N	nonexistent	—
②Summer	Kexidala	3035m	94°15'40.56"E 39°22'46.95"N	abundance	detected
③Autumn	Baozigu	2812m	93°49'06.10"E 39°24'48.92"N	nonexistent	—
④Winter	Jijigou	2715m	93°52'16.86"E 39°25'54.35"N	nonexistent	—

<table>
<tr><th>NO.</th><th>Date of birth</th><th>Sampling date and Pasture number</th><th>F1 antibody titer</th><th>Sampling date</th><th>F1 antibody titer</th></tr>
<tr><td>A20D6 mother</td><td>—</td><td rowspan="8">2020.11.20
④</td><td>1:512</td><td rowspan="8">2020.12.17</td><td>1:2048</td></tr>
<tr><td>A20D 6-1</td><td rowspan="7">2020.11.18</td><td>1:256</td><td>1:128</td></tr>
<tr><td>A20D 6-2</td><td>1:256</td><td>1:64</td></tr>
<tr><td>A20D 6-3</td><td>1:256</td><td>1:64</td></tr>
<tr><td>A20D 6-4</td><td>1:256</td><td>1:64</td></tr>
<tr><td>A20D 6-5</td><td>1:256</td><td>1:64</td></tr>
<tr><td>A20D 6-6</td><td>1:256</td><td>1:32</td></tr>
<tr><td>A20D 6-7</td><td>1:128</td><td>1:64</td></tr>
</table>

A7

Species	shepherd dog	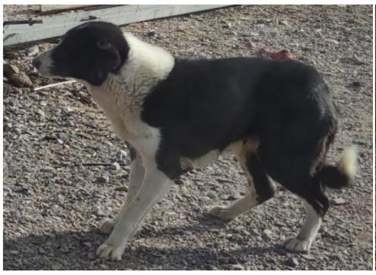 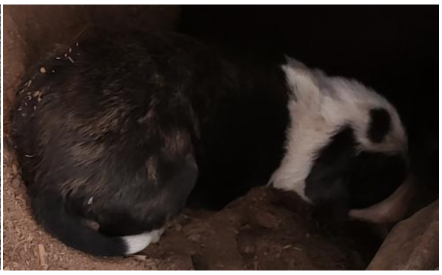
Pastures		

Seasons	Location	Altitude	Latitude and longitude	Distribution of marmot	Detection of *Y. pestis*
①Spring	Qingshuigou	2676m	94°29'25.01"E 39°28'9.80"N	nonexistent	—
②Summer	Hejinbaoquanzi	3572m	94°39'16.11"E 39°19'23.54"N	existent	detected
③Autumn	Yandantu	3654m	94°40'25.26"E 39°22'1.45"N	existent	not detected
④Winter	Dongtai	2790m	94°39'55.89"E 39°27'45.32"N	existent	not detected

NO.	Date of birth	Sampling date and Pasture number	F1 antibody titer	Sampling date	F1 antibody titer
A20D7 mother	—	2020.11.24 ③	1:256	/	
A20D7-1	2020.11.17		1:64	2020.12.16	1:32
A20D7-2			1:64		1:64
A20D7-3			1:64		1:32
A20D7-4			1:64		1:32
A20D7-5			1:32		1:32
A20D7-6			1:64		1:32
A20D7-7			1:64	/	

A8

Species	shepherd dog	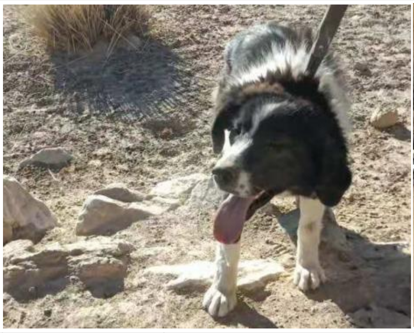 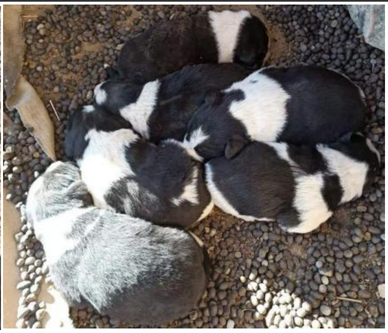
Pastures		

Seasons	Location	Altitude	Latitude and longitude	Distribution of marmot	Detection of *Y. pestis*
①Spring	Donggelieke	3162m	93°13'16.48"E 39°18'6.57"N	nonexistent	—
②Summer	Woertazala	3393m	93°21'22.98"E 39°18'5.49"N	abundance	not detected
③Autumn	Annanba	2698m	93°1'18.11"E 39°17'43.59"N	nonexistent	—
④Winter	Jianamasayi	3019m	92°52'57.67"E 39°154.06"N	nonexistent	—

NO.	Date of birth	Sampling date	F1 antibody titer	Sampling date	F1 antibody titer	Sampling date	F1 antibody titer
A20D8 mother	—	2020.12.1	1:1024	2020.12.18	1:512	2021.5.7	1:128
A20D8-1	2020.10.4		1:128		1:64		negative
A20D8-2			1:128		1:64	/	
A20D8-3			1:128		1:64	/	
A20D8-4			1:128		1:64	/	
A20D8-5			1:128		1:64	/	
A20D8-6			1:128		1:64	/	

A9

Species	shepherd dog	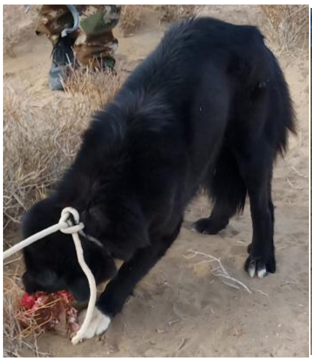 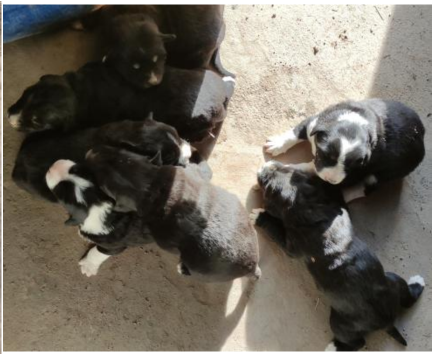
Pastures		

Seasons	Location	Altitude	Latitude and longitude	Distribution of marmot	Detection of *Y. pestis*
①Spring	Jiaerwuzong	2978m	94°29'25.11"E 39°28'9.93"N	nonexistent	—
②Summer	Jiaerma	3433m	94°38'51.92"E 39°21'36.88"N	abundance	detected
③Autumn	Jiaerma	3711m	94°35'51.76"E 39°22'1.65"N	abundance	detected
④Winter	Qunhuer	2646m	94°37'21.75"E 39°28'53.03"N	nonexistent	—

NO.	Date of birth	Sampling date	F1 antibody titer	Sampling date	F1 antibody titer	Sampling date	F1 antibody titer
A20D9 mother	—	2020.12.7	1:2048	2020.12.19	1:2048	2021.4.26	1:512
A20D9-1	2020.12.4		1:2048		1:256	2021.5.7	negative
A20D9-2			1:2048		1:256		negative
A20D9-3			1:1024		1:256	/	
A20D9-4			1:256		1:256	2021.4.26	negative
A20D9-5			1:1024		1:256	/	
A20D9-6			1:1024		1:256	/	
A20D9-7			1:1024		1:512	/	
A20D9-8		/			1:256	/	

A10

Species	shepherd dog	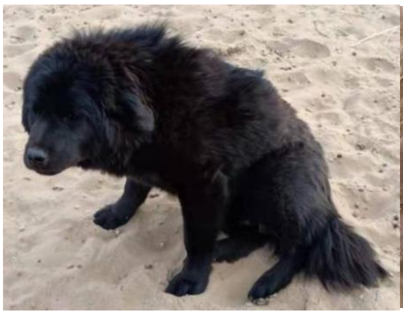 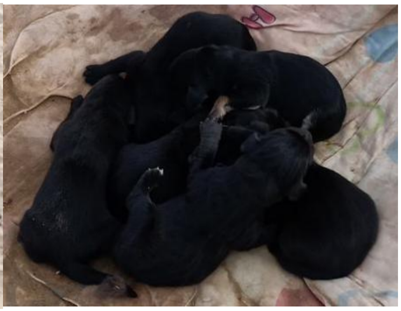
Pastures		

Seasons	Location	Altitude	Latitude and longitude	Distribution of marmot	Detection of *Y. pestis*
①Spring	Jiaerwuzong	2757m	94°29'35.64"E 39°27'39.44"N	existent	not detected
②Summer	Jiaerma	3920m	94°33'38.31"E 39°16'37.87"N	existent	not detected
③Autumn	Wugequanzi	3841m	94°31'37.88"E 39°22'0.13"N	few	not detected
④Winter	Qunhuer	2774m	94°38'12.73"E 39°28'11.96"N	nonexistent	—

NO.	Date of birth	Sampling date	F1 antibody titer	Sampling date	F1 antibody titer	Sampling date	F1 antibody titer
A20D10 mother	—	2020.12.7	1:512	2020.12.19	1:512	2021.4.26	1:64
A20D10-1	2020.12.4		1:128		1:32	2021.4.30	negative
A20D10-2			1:128		1:32	/	
A20D10-3			1:128		1:32	2021.4.26	negative
A20D10-4			1:128		1:32	/	
A20D10-5			1:128		1:32	/	
A20D10-6		/			1:32	/	

A11

NO.	Date of birth	Sampling date	F1 antibody titer
A21D11 mother	—	2021.4.26	1:64
A21D11-1	2021.2.26		negative
A21D11-2			negative

Qilian Mountain region S1

<table>
<tr><td>Species</td><td>shepherd dog</td><td rowspan="2" colspan="3">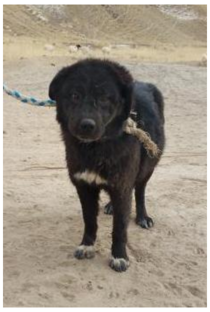</td></tr>
<tr><td colspan="2">Pastures</td></tr>
<tr><td colspan="2">Location</td><td>Altitude</td><td colspan="2">Latitude and longitude</td></tr>
<tr><td colspan="2">Jingou village, Shibaocheng</td><td>3054m</td><td colspan="2">97°11'17"E
39°38'48"N</td></tr>
<tr><td>NO.</td><td>Date of birth</td><td colspan="2">Sampling date</td><td>F1 antibody titer</td></tr>
<tr><td>S20D1 mother</td><td>—</td><td rowspan="3" colspan="2">2020.4.10</td><td>1:2048</td></tr>
<tr><td>S20D1-1</td><td rowspan="2">2020.3.23</td><td>1:64</td></tr>
<tr><td>S20D1-2</td><td>1:128</td></tr>
</table>

S2

<table>
<tr><td>Species</td><td>shepherd dog</td><td rowspan="2" colspan="3">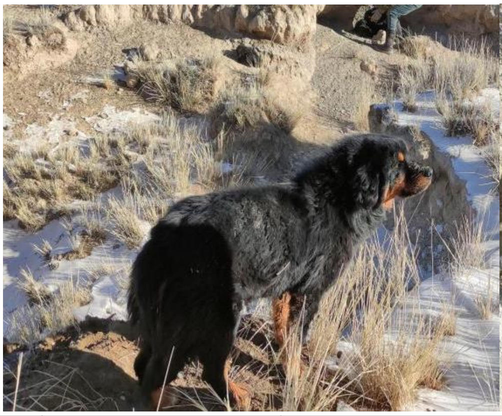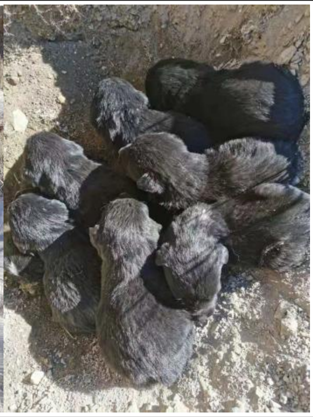</td></tr>
<tr><td colspan="2">Pastures</td></tr>
<tr><td colspan="2">Location</td><td>Altitude</td><td colspan="2">Latitude and longitude</td></tr>
<tr><td colspan="2">Haobulege village,
Dangchengwan town</td><td>2900m</td><td colspan="2">95°14'7103"E
39°66'0202"N</td></tr>
<tr><td>NO.</td><td>Date of birth</td><td colspan="2">Sampling date</td><td>F1 antibody titer</td></tr>
<tr><td>S21D2 mother</td><td>—</td><td rowspan="6" colspan="2">2021.1.9</td><td>1:64</td></tr>
<tr><td>S21D2-1</td><td rowspan="5">2020.12.17</td><td>1:16</td></tr>
<tr><td>S21D2-2</td><td>1:16</td></tr>
<tr><td>S21D2-3</td><td>1:16</td></tr>
<tr><td>S21D2-4</td><td>1:16</td></tr>
<tr><td>S21D2-5</td><td>1:16</td></tr>
</table>

S3

<table>
<tr><td>Species</td><td>shepherd dog</td><td colspan="8" rowspan="2">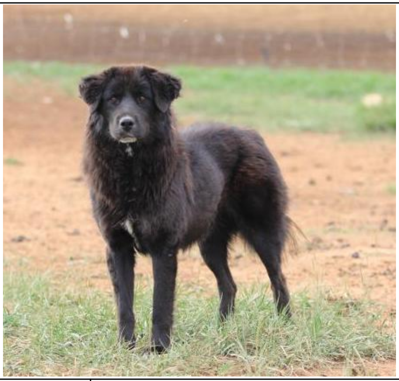</td></tr>
<tr><td colspan="2">Pastures</td></tr>
<tr><td colspan="3">Location</td><td colspan="2">Altitude</td><td colspan="5">Latitude and longitude</td></tr>
<tr><td colspan="3">Jingou village, Shibaocheng</td><td colspan="2">2873m</td><td colspan="5">97°13'5757"E
39°40'0834"N</td></tr>
<tr><td>NO.</td><td>Date of birth</td><td>Sampling date</td><td>F1 antibody titer</td><td>Sampling date</td><td>F1 antibody titer</td><td>Sampling date</td><td>F1 antibody titer</td><td>Samp-ling date</td><td>F1 antibody titer</td></tr>
<tr><td>S21D3 mother</td><td>—</td><td rowspan="2">2021.1.13</td><td>1:256</td><td colspan="2">/</td><td rowspan="2">2021.7.11</td><td>1:128</td><td>2021.10.18</td><td>1:64</td></tr>
<tr><td>S21D3-1</td><td>2020.10.11</td><td>1:32</td><td>2021.4.14</td><td>negative</td><td>1:16</td><td colspan="2">/</td></tr>
<tr><td>S21D3-2</td><td rowspan="2">2021.9.1</td><td colspan="2">/</td><td colspan="2">/</td><td colspan="2">/</td><td rowspan="2">2021.10.1</td><td>negative</td></tr>
<tr><td>S21D3-3</td><td colspan="2">/</td><td colspan="2">/</td><td colspan="2">/</td><td>negative</td></tr>
</table>

S4

<table>
<tr><td>Species</td><td>shepherd dog</td><td colspan="4" rowspan="2">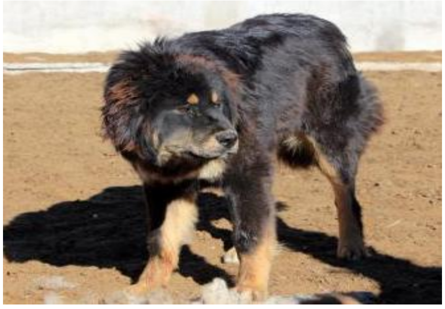</td></tr>
<tr><td colspan="2">Pastures</td></tr>
<tr><td colspan="2">Location</td><td colspan="2">Altitude</td><td colspan="2">Latitude and longitude</td></tr>
<tr><td colspan="2">Jingou village, Shibaocheng</td><td colspan="2">2761m</td><td colspan="2">97°13'1375"E
39°41'5407"N</td></tr>
<tr><td>NO.</td><td colspan="2">Date of birth</td><td colspan="2">Sampling date</td><td>F1 antibody titer</td></tr>
<tr><td>S21D4 mother</td><td colspan="2">—</td><td colspan="2" rowspan="2">2021.1.13</td><td>1:128</td></tr>
<tr><td>S21D4-1</td><td colspan="2">2020.11.19</td><td>1:16</td></tr>
</table>

S5

Species	shepherd dog	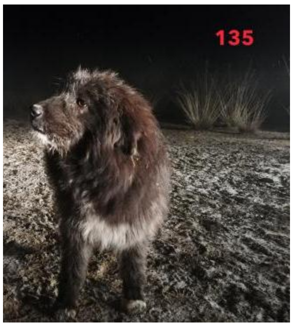
Pastures		

Location	Altitude	Latitude and longitude
Jingou village, Shibaocheng	3053m	97°12'55"E<br>39°39'21"N

NO.	Date of birth	Sampling date	F1 antibody titer
S21D5 mother	—	2021.1.14	1:64
S21D5-1	2020.12.8		1:16

S6

Species	shepherd dog	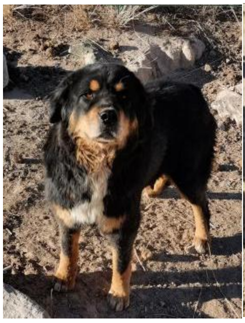 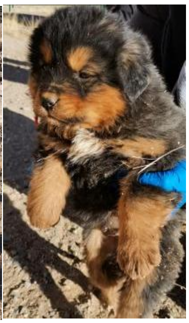
Pastures		

Location	Altitude	Latitude and longitude
Jiaerwuzong	2757m	94°29'35.64"E<br>39°27'39.44"N

NO.	Date of birth	Sampling date	F1 antibody titer	Sampling date	F1 antibody titer	Sampling date	F1 antibody titer
S21D6 mother	—	2021.1.17	1:512	2021.4.9	1:256	2021.7.13	1:128
S21D6-1	2020.11.26		1:16		negative		negative
S21D6-2			1:16		negative	/	

S7

NO.	Date of birth	Sampling date	F1 antibody titer
S21D7 mother	—	2021.4.11	1:64
S21D7-1	2020.12.27		negative
S21D7-2			negative
